# BBSNet: An Intelligent Grading Method for Pork Freshness Based on Few-Shot Learning

**DOI:** 10.3390/foods14142480

**Published:** 2025-07-15

**Authors:** Chao Liu, Jiayu Zhang, Kunjie Chen, Jichao Huang

**Affiliations:** 1 College of Engineering, Nanjing Agricultural University, Nanjing 210000, China; lucho1985@163.com (C.L.); 2020212020@stu.njau.edu.cn (J.Z.); 2 College of Intelligent Manufacturing, Taizhou Institute of Science and Technology Nanjing University of Science and Technology, Taizhou 225300, China

**Keywords:** pork freshness, few-shot learning, BiFormer, fine-tuning

## Abstract

Deep learning approaches for pork freshness grading typically require large datasets, which limits their practical application due to the high costs associated with data collection. To address this challenge, we propose BBSNet, a lightweight few-shot learning model designed for accurate freshness classification with a limited number of images. BBSNet incorporates a batch channel normalization (BCN) layer to enhance feature distinguishability and employs BiFormer for optimized fine-grained feature extraction. Trained on a dataset of 600 pork images graded by microbial cell concentration, BBSNet achieved an average accuracy of 96.36% in a challenging 5-way 80-shot task. This approach significantly reduces data dependency while maintaining high accuracy, presenting a viable solution for cost-effective real-time pork quality monitoring. This work introduces a novel framework that connects laboratory freshness indicators to industrial applications in data-scarce conditions. Future research will investigate its extension to various food types and optimization for deployment on portable devices.

## 1. Introduction

With the improvement of living standards, consumer demand for high-quality meat, particularly with an emphasis on freshness, is increasing. Current methods for assessing pork freshness primarily rely on physicochemical analyses, including microbial counts [1], total volatile basic nitrogen (TVB-N) detection [2], and pH measurement [3]. Although these methods are accurate and reliable, they are time-consuming and require destructive sample processing, making them entirely unsuitable for the rapid and online detection of pork freshness [4]. Non-destructive alternatives utilizing spectral imaging and electronic nose (e-nose) technologies have emerged as significant tools in food quality assessment [5,6]. The combination of hyperspectral imaging (400–1000 nm) with least squares support vector machines (LS-SVMs) has demonstrated effectiveness in predicting TVB-N levels [7]. Additionally, fluorescence hyperspectral imaging, paired with partial least squares regression (PLSR), has been successfully applied to evaluate the quality of frozen pork [8]. However, challenges such as spectral overlap and the selective effectiveness of certain wavelengths limit the accuracy of feature extraction. Similarly, e-nose systems that employ techniques such as linear discriminant analysis (LDA) [9] or sensor arrays analyzed through principal component analysis (PCA) [10] encounter issues related to high costs and operational complexity, despite their promising levels of accuracy [11]. Multimodal approaches that integrate spectral imaging and e-noses [12,13,14] enhance detection robustness; however, they require dedicated hardware systems that are prone to inherent time delays and data synchronization errors. Conversely, computer vision provides rapid, non-destructive solutions, achieving accuracies of 92.5% in grading pork color and marbling when using support vector machines (SVM) [15], and facilitating intramuscular fat estimation through gradient boosting machines [16]. Advanced image processing techniques, particularly attention-enhanced U-Net models, have further optimized feature extraction [17].

Nevertheless, deep learning models that rely on image processing exhibit a significant dependency on dataset size [18,19]. Insufficiently large datasets hinder the model’s ability to adequately capture the characteristic variations inherent to each category. While data augmentation can artificially increase sample sizes, evaluations of pork freshness based on visual attributes (color, texture, luster) suggest that augmentation does not fundamentally enhance the critical characteristics intrinsic to different freshness levels. This limitation significantly constrains the generalization capability of such models [20].

Few-shot learning (FSL) presents a promising alternative for effective classification with minimal annotated data [21]. Pan et al. [22] utilized Faster R-CNN in conjunction with FSL to assess the severity of strawberry leaf scorch, achieving a maximum recognition accuracy of 96.67% with only 550 training samples. Similarly, Villon et al. [23] employed FSL to classify underwater images of 20 coral reef fish species, attaining an accuracy of 93.93% with just 30 training samples per category. Collecting a large and accurately annotated dataset for pork information necessitates significant effort. The use of minimal samples for image classification is particularly critical for tasks such as pork freshness grading. FSL can incorporate biological prior knowledge (e.g., myoglobin oxidation kinetics, microbial growth curves) as meta-learning constraints, thereby reducing dependence on purely empirical data. Moreover, it enables the model to be initialized with a minimal number of labeled samples through cross-domain feature transfer from data-rich food categories to pork [24].

These research findings demonstrate that few-shot learning methods can achieve significantly superior results compared to classical deep learning approaches, particularly when annotated image data is severely limited. Despite these advances across various domains, the application of few-shot learning techniques to the challenge of pork freshness grading remains unexplored.

Our contributions include the following:

(i) the development of BBSNet, a lightweight architecture that integrates dynamic attention mechanisms and channel normalization, achieving an accuracy of 96.36% in 5-way 80-shot tasks. This approach addresses the high data dependency characteristic of traditional deep learning methods in pork freshness detection.

(ii) The replacement of batch normalization in ShuffleNetV2 and layer normalization in BiFormer with BCN, which enhances feature stability and discriminability, resulting in a 6.27% improvement in 5-way 5-shot accuracy compared to baseline models.

(iii) The performance of BBSNet surpasses that of classic few-shot models (MAML, prototypical networks) and CNNs such as AlexNet and ResNet50 under conditions of limited data. BBSNet achieved accuracies of 59.72% (1-shot) and 78.84% (5-shot) in 5-way tasks, demonstrating its adaptability to scenarios with scarce labeled data.

The remainder of the paper is structured as follows: Section 2 details the materials and methods, including dataset acquisition, the few-shot learning method based on BBSNet, model training, and evaluation metrics. Section 3 presents the results and discussions, comparing the performance of various models and analyzing the impacts of the BCN layer, the BiFormer attention mechanism, and the number of support set samples. Section 4 summarizes the findings of the entire paper and provides an outlook on future work.

## 2. Materials and Methods

### 2.1. Data Set Acquisition

#### 2.1.1. Pork Freshness Grading Criteria

Total colony count is a critical indicator of meat spoilage, serving as a determinant for the continued consumption of pork. Additionally, positive correlations have been observed among pork color, luster, and other quality attributes [25]. Consequently, microbial colony concentration was utilized as a measure of pork freshness in this study.

Pork samples were obtained from the Jiangsu Taizhou RT-Mart Supermarket. The hind leg meat was cut into pieces measuring approximately 50 mm × 80 mm with a thickness of 10 mm, resulting in a total of 500 slices. These slices were then packaged in sterilized self-sealing bags and stored in a refrigerator at 4 °C for durations of 0, 24, 48, 72, and 96 h. Subsequently, the microbial concentrations of the pork samples were determined in accordance with the GB 47892-2016 standard [26], “Determination of Microbial Counts in Foods.” The microbial colony concentrations of the pork samples were measured separately, and the average value was calculated to represent the microbial colony concentration parameter for the samples. According to the national standard GB/T 9959.2-2008 [27], which pertains to split fresh and frozen lean pork, the total number of bacterial colonies in fresh meat should not exceed 10^6^ CFU/g. After 72 h of storage at 4 °C, the total colony count of the sampled pork reached 1.778279 × 10^6^ CFU/g, surpassing the standard limit for fresh pork total colony count. Consequently, pork freshness was classified into five grades based on microbial colony concentration parameters, as summarized in Table 1. This grading system closely aligns with the findings of Zhang et al. (2023) [11] and Cheng et al. (2024) [14].

#### 2.1.2. Pork Freshness Dataset

Five pork samples, each representing different freshness grades, were imaged under natural light using a CCD camera (DH-HV2031UC-Z™, Dahua Technology, Hangzhou, China) with a resolution of 4.2 megapixels and global shutter technology. The captured images were subsequently uploaded to a computer via USB for storage. A total of 120 samples were collected for each grade of pork freshness, resulting in an overall dataset of 600 samples. These images were resized to 224 × 224 pixels for subsequent analysis. The images corresponding to various pork freshness classes are illustrated in Table 2. First-grade and second-grade fresh pork samples exhibited a bright red color with good luster, while third-grade fresh pork samples displayed a dark red hue with an average appearance. In contrast, first-grade and second-grade spoiled pork samples were characterized by a dark red color and poor luster.

A k-way, n-shot task was established, where k denotes the number of categories and n indicates the number of samples in the support set. In this study, pork freshness was categorized into five classes; thus, the research task is defined as a 5-way n-shot task. To validate the model’s performance, n images are randomly selected from each class of the dataset to form the support set.

### 2.2. Few-Shot Learning Method Based on BBSNet

In few-shot learning, images are mapped to task-specific metric spaces to facilitate similarity-based recognition. Prototypical networks [28] are noted for their simplicity and efficiency. Recent studies have incorporated self-attention mechanisms [29,30,31] to improve feature discrimination. These works demonstrate that attention-driven prototype refinement has the potential to mitigate feature ambiguity in scenarios with limited data.

**Figure 1:**
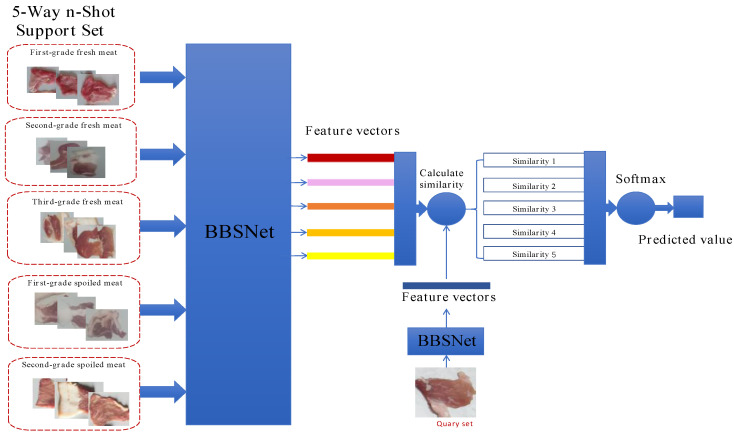
Basic structure of few-shot learning.

To address the high computational costs associated with existing attention-enhanced prototypical networks, we propose BBSNet, a lightweight architecture designed for five-class pork freshness classification. Built upon prototypical networks, BBSNet utilizes the ShuffleNetV2 backbone to minimize computational load while integrating the BiFormer module to enhance feature discriminability. Additionally, the BCN normalization method is employed to facilitate stable training with higher learning rates and reduced dependency on initialization. The proposed pipeline involves the following steps:

(i) Extracting feature vectors using BBSNet;

(ii) Computing cosine similarities between query features and the five class prototypes in the support set;

(iii) Determining the category with the highest similarity through softmax activation. This design effectively balances efficiency and accuracy, as illustrated in Figure 1.

#### 2.2.1. Composition of BBSNet

Metric-based methods typically employ episodic training strategies to train the feature extractor, utilizing either a fixed or parameterized distance metric. This approach places substantial demands on the performance of the feature extraction network. A high-performing feature extraction network is crucial for ensuring the accuracy of metric operations in few-shot learning [32,33].

**Figure 2:**
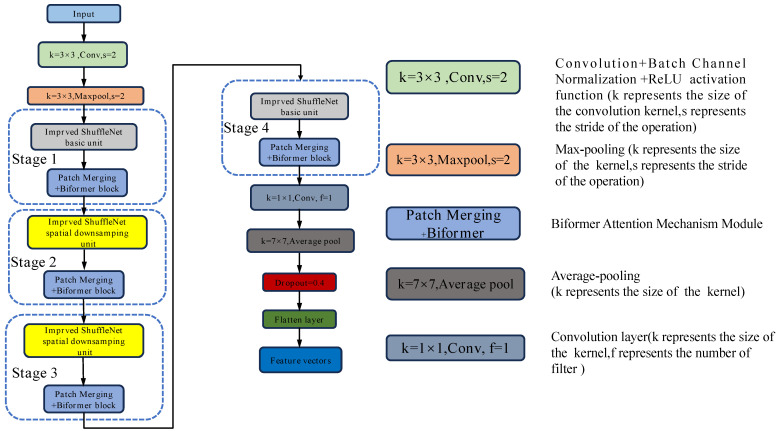
Basic structure of BBSNet.

Figure 2 illustrates the structure of the BBSNet feature extraction network. The input image is processed through a convolutional layer with a 3 × 3 convolutional kernel and a stride of 2, before entering the first stage after passing through a max pooling layer. This stage comprises the basic unit of ShuffleNetV2 in conjunction with the patch merging + BiFormer block module. The second through fourth stages consist of the ShuffleNetV2 downsampling unit alongside the patch merging + BiFormer block module. After the original image undergoes processing through four computational stages, it is subsequently passed through a convolutional layer that employs 11 convolutional kernels with a filter size of 1. This is followed by a global average pooling layer, a dropout layer with a dropout rate of 0.4, and a flatten layer. Consequently, the image features are extracted into a one-dimensional vector. Dropout is utilized for model regularization by randomly deactivating a portion of the neurons, thereby enhancing network sparsity, which is beneficial for feature selection and the prevention of overfitting during training [11]. The Adam algorithm is employed as the model optimizer for the feature extraction network, with the learning rate set to 0.001.

#### 2.2.2. Upgrading of ShuffleNetV2 Module

Although ShuffleNetV2 prioritizes computational efficiency, its effectiveness in fine-grained tasks, such as pork freshness grading, is limited by insufficient feature discrimination. To address this limitation, we enhance the network architecture through two structural innovations.

(a) BCN:

Integrated after each convolutional layer (3 × 3 depthwise and 1 × 1), BCN, in conjunction with ReLU, stabilizes feature distributions while preserving discriminative texture and color details.

(b) BiFormer-enhanced dual-branch design:

Basic unit: Features are processed sequentially through depthwise convolution → BCN → patch merging → BiFormer (capturing spatial-channel dependencies) → channel shuffling.

Downsampling unit: Parallel branches reduce spatial resolution by half. The right branch incorporates BiFormer and BCN to facilitate multi-scale feature fusion.

**Figure 3:**
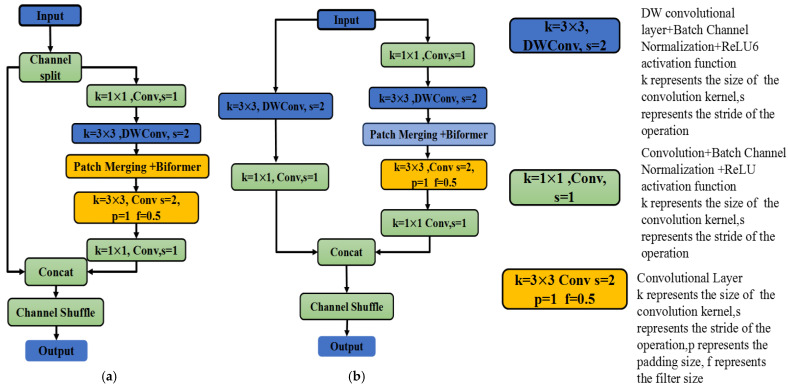
(**a**) Improved ShuffleNetv2, basic unit; (**b**) Improved ShuffleNetv2, spatial downsampling unit.

The improved ShuffleNetv2 basic unit and spatial downsampling unit structures are illustrated in Figure 3.

Given the sensitivity of local feature differences, such as color and texture, in images of pork freshness, BCN effectively preserves subtle feature variations across different regions. This is achieved by performing local statistical normalization on image blocks, thereby avoiding the potential blurring of local information that may result from the global normalization approach of BN. The BiFormer module optimizes computational efficiency without compromising feature specificity. Collectively, these modifications strengthen ShuffleNetV2’s ability to discern critical freshness-related patterns in pork images, aligning with the precision requirements of food quality assessment [34].

#### 2.2.3. Accelerating Feature Fitting with Batch Channel Normalization

Internal variable shifts may occur due to the randomness in parameter initialization and variations in input data [35]. BCN integrates the advantages of BN [36] and LN [37] by utilizing the correlations between channels and batch processing.

To address the limitations of traditional BN and LN in capturing both spatial and channel-wise statistics, we adopted BCN proposed by Khaled et al. (2023) [38]. BCN dynamically balances the contributions of batch-wise and channel-wise normalization through a learnable parameter ι, enabling adaptive feature scaling and shifting.

The BCN operation is defined as follows:

(1) $$Y=\gamma \left[\iota \frac{\left(x_{i}-\mu_{2}\right)}{\sqrt{\sigma_{1}^{2}+\varepsilon }}+(1-\iota )\frac{\left(x_{i}-\mu_{2}\right)}{\sqrt{\sigma_{2}^{2}+\varepsilon }}\right]+\beta$$

where xi is the input image tensor; Ɩ, γ, and β are learnable parameters; and ɛ is a small constant used for numerical stability. By adopting the BCN method, all channels in the convolutional layer shared the same normalization terms μ and σ^2^, μ_1_, μ_2_ and σ_1_^2^, σ^2^_2_ denote the mean and variance computed across both batch and channel dimensions. This improved model performance in deep learning networks [39]. In this research, the LN layers in the BiFormer module and the BN layers in ShuffleNet were all replaced with BCN. Here, the learnable parameters Ɩ, γ, and β were all randomly initialized from a normal distribution (with a mean of 0 and standard deviation of 1).

#### 2.2.4. Upgrading of BiFormer Module

**Figure 4:**
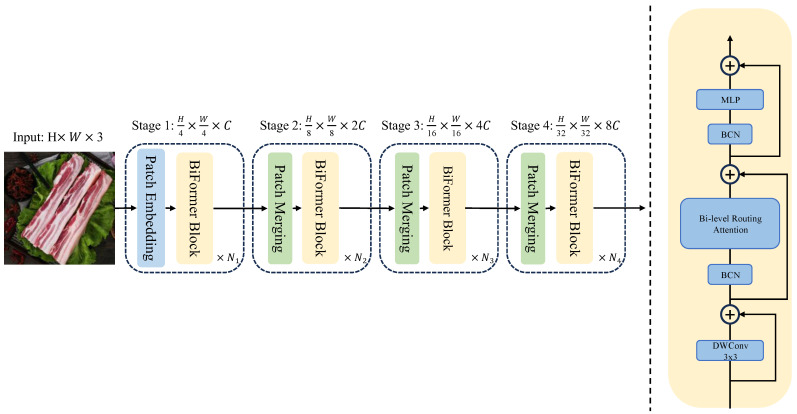
Structure of BCN-BiFormer.

In recent years, vision transformers have made significant advancements in the field of computer vision [40,41]. The existing BiFormer module [42] incorporates an LN layer, which necessitates the computation of a global dimension for each sample, resulting in substantial computational costs. Furthermore, LN demonstrates insensitivity to variations in sequence length, rendering it more appropriate for sequence-based tasks such as natural language processing [37]. To mitigate these limitations, this study substitutes the LN layer with a BCN layer, thereby constructing a BCN-BiFormer attention mechanism, as illustrated in Figure 4. The BiFormer module comprises a 3 × 3 depthwise convolutional layer, a bi-level routing attention layer, a BCN normalization layer, and a dilated multi-layer perceptron (with a dilation ratio of e). The input consists of an H × W × 3 three-channel image, which is processed through four stages:

Stage 1: An overlapping patch embedding layer and the BCN-BiFormer module reduce the feature size to H/4 × W/4 × 3.

Stages 2–4: Block merging modules and BCN-BiFormer modules halve the spatial dimensions while doubling the channel count at each stage.

The DWConv denotes depthwise convolution, the BCN represents batch channel normalization processing, and the MLP refers to a multilayer perceptron.Structure of BCN-BiFormer is illustrated in Figure 4 The derivation of the output vector formula for the BiFormer attention mechanism is provided in Appendix A.

In the image classification task, the expansion ratio (e) of the MLP was set to 3, the parameter S was set to 7, and the top-k values in the four stages of BRA were specified as 7, 8, 16, and 49, respectively [42].

#### 2.2.5. Probability Distribution Function Based on Cosine Similarity

As detailed in Section 2.1.2, the core challenge in recognizing pork freshness lies in capturing subtle differences in image features, which are primarily influenced by variations in color and luster. Experimental observations indicate that while storage time affects color intensity—such as a deepening of redness—and reduces surface luster (thereby impacting pixel brightness), the geometric orientation (direction) of feature vectors derived from the same freshness class remains relatively stable. For instance, texture patterns and color distribution gradients, which are key indicators of freshness, maintain consistent directional relationships across images of the same class, despite fluctuations in overall brightness (vector magnitude) caused by changes in luster (refer to Table 2 in Section 2.1.2). Cosine similarity [43] is particularly well-suited for this scenario, as it quantifies the angular difference between feature vectors. This metric emphasizes the consistency of direction rather than the magnitude of the vectors, making it an effective measure for comparing similarity in high-dimensional spaces.

This aligns precisely with the characteristics of our dataset: while variations in luster lead to significant discrepancies in vector norms—rendering metrics such as Euclidean distance unreliable due to their amplification of magnitude disparities—the discriminative information pertinent to freshness classification is found in the directional nuances of color and texture features. For instance, the gradient of color transition from muscle to fat tissue serves as a critical indicator.

**Figure 5:**
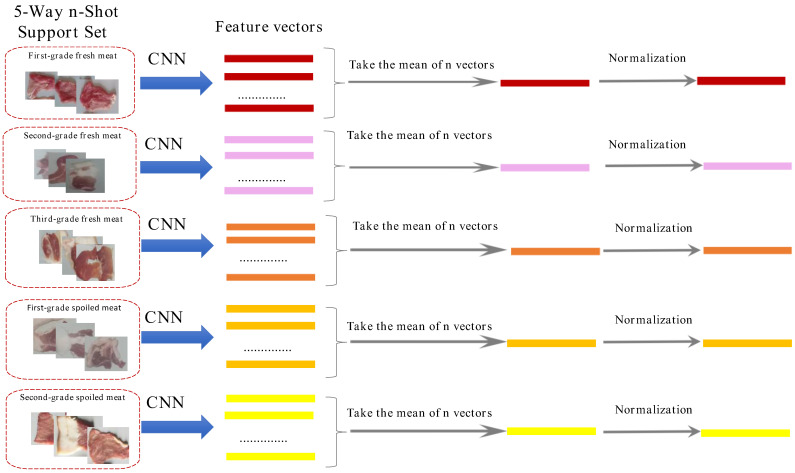
Few-shot learning feature vector extraction and processing.

The n feature vectors were averaged and subsequently normalized to yield five one-dimensional feature vectors: $\mu_{1},\;\mu_{2},\;\mu_{3},\;\mu_{4},\;\mu_{5}$. Figure 5 illustrates the calculation process. The vector q was derived following the feature extraction and normalization of each image in the query set using the same convolutional neural network.

**Figure 6:**
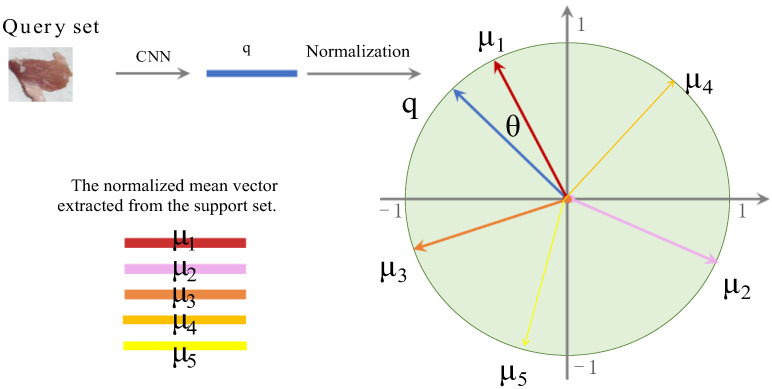
Cosine similarity comparison.

After the normalization processing of query and support set image vectors, cosine similarity values among the vector q and $\mu_{1},\mu_{2},\mu_{3},\mu_{4},\mu_{5}$ were compared. As presented in Figure 6, cosine similarity value was denoted as $sim\left(\mu_{n},q\right)$. Here, θ is the angle between the two vectors being compared, and the smaller the value of θ, the greater the similarity between the two vectors. Cosine similarity was expressed as follows:

(2) $$sim\left(\mu_{n},q\right)=\frac{\mu_{n}q}{{\left\|\mu_{n}\right\|}_{2}\cdot {\left\|q\right\|}_{2}}=\cos\theta$$

where $\mu_{n}$ is the mean vector of the nth support set, ${\left\|\mu_{n}\right\|}_{2}$ is the 2-norm of the vector $\mu_{n}$, and ${\left\|q\right\|}_{2}$ is the 2-norm of the vector q.

A softmax classifier was applied to predict the similarity between support and query sets.

(3) p=softmax(Mq)

where M is the mean vectors of the samples of the five pork freshness categories in the support set, defined as

(4) $$M={\left[\mu_{1}^{T},\mu_{2}^{T},\mu_{3}^{T},\mu_{4}^{T},\mu_{5}^{T}\right]}^{T}$$

and q is the feature vector of the query set. Then, the probability distribution p of the query set samples was defined as

(5) $$p=soft\max{\left(\mu_{1}^{T}q,\mu_{2}^{T}q,\mu_{3}^{T}q,\mu_{4}^{T}q,\mu_{5}^{T}q\right)}^{T}$$

### 2.3. Fine-Tuning Strategy

This study presents a cosine similarity-based fine-tuning framework that incorporates an adaptive softmax classifier to enhance few-shot recognition. After pre-training the BBSNet feature extractor on support set samples, both support and query images were encoded into one-dimensional feature vectors. The cosine similarities between these vectors were processed through a softmax classifier, whose parameters were optimized using support set data to align the weight vectors with intrinsic feature similarity patterns. To maintain computational efficiency, the pre-trained BBSNet parameters remained frozen during the fine-tuning process. The optimization employed a two-stage strategy for each epoch:

(a) Cross-entropy minimization on support set samples refines the classification boundaries; (b) entropy regularization applied to query set features mitigates overfitting and enhances discriminability.

This dual mechanism synergistically improves model generalizability, resulting in significant accuracy gains in few-shot tasks while preserving computational efficiency.

#### 2.3.1. Updating Cross-Entropy Loss Function

To distinguish between model training samples and fine-tuning samples, during fine-tuning process, the samples and labels in support set were denoted as $\left(x_{j},y_{j}\right)$. Here, $f\left(x_{j}\right)$ is the feature vector extracted through BBSNet and pj is the predicted label of the model, defined as

(6) $$p_{j}=soft\max\left[W\cdot f\left(x_{j}\right)+b\right]$$

As described in Section 2.2, to accelerate fine-tuning convergence speed, the value of W was initialized to M and b was set as a zero column vector. Here, M is the mean of the feature vectors in the support set.

(7) $$W=M={\left[\mu_{1}^{T},\mu_{2}^{T},\mu_{3}^{T},\mu_{4}^{T},\mu_{5}^{T}\right]}^{T}$$

(8) $$b={\left[0,0,0,0,0\right]}^{T}$$

During fine-tuning process, support set samples were applied as training samples and the cross-entropy loss function was used to update the values of W and b. Cross-entropy loss function was calculated using Equation (9), where y_j_ is true label and p_j_ is predicted label.

(9) $$loss=\min\sum_{j}CrossEntropy(y_{j},p_{j})=-\sum_{j=1}^{n}y_{j}\log P(f(x_{j})$$

#### 2.3.2. Updating Entropy Regularization Function

During the updating process of the cross-entropy loss function, due to the small number of training samples, the model was prone to overfitting. In this research, entropy regularization was added to the cross-entropy loss function to prevent overfitting.

An image in query set was denoted as A. The feature vector extracted by BBSnet was denoted as f(A). Probability was calculated using a softmax activation function as a probability distribution $p=soft\max\left[W\cdot f\left(A\right)+b\right]$. Assuming that there were m samples in the query set, the entropy regularization term of the m samples was denoted as H(p):

(10) $$H(p)=-\sum_{i=1}^{m}p_{i}\log p_{i}$$

where i is the number of samples in query set. H(p) measures the amount of probability distribution p information. Substituting Equation (10) into Equation (9), the loss function in fine-tuning process was obtained as follows:

(11) $$loss=-\sum_{j=1}^{n}y_{j}\log P\left(f(x_{j})\right)+\left(-\sum_{i=1}^{m}p_{i}\log p_{i}\right)$$

By incorporating the entropy regularization term of the query set into the loss function, the optimizer subtracts this regularization term in each epoch. When the regularization term is large, a significant value is deducted in each epoch, thereby accelerating the convergence speed of the loss function.

### 2.4. Model Training

#### 2.4.1. Experimental Design

The model was compiled in Windows environment on computer with I7-8700 CPU, 8G of memory, and NVIDIA GTX1060 6G graphics card. All the code was implemented using Keras framework based on TensorFlow 2.0 version.

To ensure a robust evaluation of small-sample models, particularly given the heightened bias risks associated with data scarcity, we employed stratified 5-fold cross-validation. The pork freshness dataset was divided into five mutually exclusive subsets, with class ratios that preserved the original distribution. In each iteration, four folds were utilized for training while one fold was reserved for testing, and this process was repeated five times to ensure comprehensive evaluation across all subsets. Multiple independent runs were conducted to yield standard deviations of accuracy, which were subsequently used for calculating t-statistics and *p*-values. A two-sample independent *t*-test, assuming equal variances, was performed to compare the mean accuracy of the proposed model against baseline models, with statistical significance established at α = 0.05 (*p* < 0.05).

#### 2.4.2. Pre-Training Setting

The mini-ImageNet dataset is widely utilized in the field of few-shot image recognition. It comprises 100 categories, featuring images of various objects such as fish and birds. Few-shot learning aims to classify unknown images by leveraging the characteristics of known images. Consequently, in accordance with classification principles, mini-ImageNet has been partitioned into a training set, a test set, and a validation set, as detailed in Table 3.

The few-shot feature extraction network was pre-trained using a transfer learning method [15] to obtain the initial model weights. To accomplish the classification task involving 100 categories of images from the mini-ImageNet dataset, the flatten layer of the convolutional neural network model was removed during pre-training. The fully connected layer utilized 100 neurons for output. The Adam optimizer was employed during the backpropagation process, with the cross-entropy loss function applied and the softmax function used for classification. The learning rate was set to 0.0001. The initial weights of BBSNet were obtained after completing 40 epochs.

### 2.5. Model Evaluation Metrics

Model evaluation parameters included true positives (TP), true negatives (TN), false positives (FP), and false negatives (FN) [44]. In this research, accuracy (Acc), sensitivity (Sen), specificity (Spe), and precision (Pre) were adopted as model evaluation metrics. Specific calculation equations were expressed as Equations (12)–(15):

(12) $$AverageAcc=\frac{\sum_{i}^{l}\frac{TP_{i}+TN_{i}}{TP_{i}+TN_{i}+FP_{i}+FN_{i}}}{l}$$

(13) $$AverageSen=\frac{\sum_{i}^{l}\frac{TP_{i}}{TP_{i}+FN_{i}}}{l}$$

(14) $$AverageSpe=\frac{\sum_{i}^{l}\frac{TN_{i}}{FP_{i}+TN_{i}}}{l}$$

(15) $$Average\mathrm{Pr}e=\frac{\sum_{i}^{l}\frac{TP_{i}}{TP_{i}+FP_{i}}}{l}$$

where TP is the number of positive samples correctly classified as positive, TN is the number of negative samples correctly classified as negative, FP is the number of negative samples incorrectly classified as positive, FN is the number of positive samples incorrectly classified as negative, l is the total number of sample categories, and i is evaluation metric for each category.

## 3. Results and Discussions

### 3.1. Performance Comparison with Classic Algorithms

#### 3.1.1. Comparison with Classic Few-Shot Models

To evaluate the performance of the few-shot model developed in this research, a comparative experiment was conducted between our model and several classic few-shot models, including MAML [45], matching networks [46], prototypical networks [28], and relation networks [47]. All models underwent pre-training using the Mini-ImageNet dataset, and their performance was assessed on two tasks: 5-way 1-shot and 5-way 5-shot learning. The results are summarized in Table 4.

The results presented in Table 4 demonstrate that BBSNet significantly outperformed traditional few-shot learning models in detecting pork freshness. In the 5-way, 1-shot scenario, BBSNet achieved an accuracy of 59.72 ± 0.98%, surpassing Matching Nets (48.12 ± 1.25%), prototypical networks (44.56 ± 1.37%), and relation networks (51.44 ± 1.21%). The performance gap is statistically significant, with t-values ranging from −16.58 to −22.01 and *p* < 0.001 for all comparisons. In the 5-way, 5-shot setting, BBSNet further improves its accuracy to 78.84 ± 0.87%, outperforming the second-best model (Matching Nets, 67.20 ± 1.18%) by 11.64%. The t-values for comparisons against prototypical networks are −20.87 (*p* < 0.001) for 1-shot and −43.15 (*p* < 0.001) for 5-shot scenarios. Notably, BBSNet exhibits the lowest standard deviation (0.87–0.98%) across all metrics, highlighting its stability in small-sample scenarios. This contrasts with traditional models, which show higher variance (1.16–1.42%), indicating BBSNet’s robustness against sample fluctuations. Our findings align with the broader literature on few-shot learning. For instance, prototypical networks (with a ResNet-18 backbone) typically struggle with intra-class variation in 1-shot settings, as reflected in our results (44.56% vs. BBSNet’s 59.72%). Although Matching Nets’s cosine similarity-based approach performs better than Euclidean distance, it still lags behind BBSNet, likely due to its heavier ResNet-18 backbone compared to BBSNet’s lightweight ShuffleNetV2. The 11.64% accuracy gap in 5-shot scenarios exceeds the typical 5–8% improvements reported in ImageNet-based few-shot studies, underscoring BBSNet’s efficiency in small-sample food quality assessment. Furthermore, relation networks’ performance (51.44% in 1-shot) highlights the challenge of learning similarity metrics from limited data, whereas BBSNet’s pre-defined cosine similarity avoids this optimization burden, leading to faster convergence.

#### 3.1.2. Comparison Against Classical Universality Algorithms

Several classic deep learning network models, including AlexNet [48], VGG16 [49], GoogLeNet [50], and ResNet50 [51], were selected to predict pork freshness. During the training process of the few-shot model, a 5-way, 80-shot method was adopted following the preset pre-training; specifically, for a 5-class classification task, 80 images were provided for each category as training samples. All models utilized 400 images as training samples. All input sample images are of size 224 × 224 × 3, with a batch size of 2. The test results are presented in Table 5.

The results demonstrated BBSNet’s significant superiority, achieving an accuracy of 96.36 ± 0.82%, which surpassed all the other models. For instance, AlexNet and ResNet50 achieved only 52.13 ± 1.78% and 68.43 ± 1.26%, respectively. BBSNet’s lowest standard deviation (0.82%) underscored its robustness in small-sample scenarios, where traditional models exhibited high variance (1.26–1.78%). Statistical analysis through one-sample *t*-tests confirmed extremely significant differences (*p* < 0.001) between BBSNet and all baseline models, with t-values ranging from −37.21 (ResNet50) to −60.92 (AlexNet).

Notably, GoogLeNet’s multi-branch architecture achieves a moderate accuracy of 58.24%. However, its lack of adaptive attention limits feature selectivity when compared to BBSNet. Similarly, AlexNet and VGG16, which utilize shallow and deep convolutional stacks, respectively, are unable to effectively capture the fine-grained texture and color changes that are indicative of pork freshness. This observation underscores the necessity of specialized designs for small-sample visual tasks. These findings are consistent with prior research indicating that attention-based networks and channel-wise normalization enhance discriminative power in image analysis. The accuracy gap of 27.93–44.23% between BBSNet and traditional models highlights a significant challenge in food quality control: achieving high-performance detection with limited labeled data.

**Figure 7:**
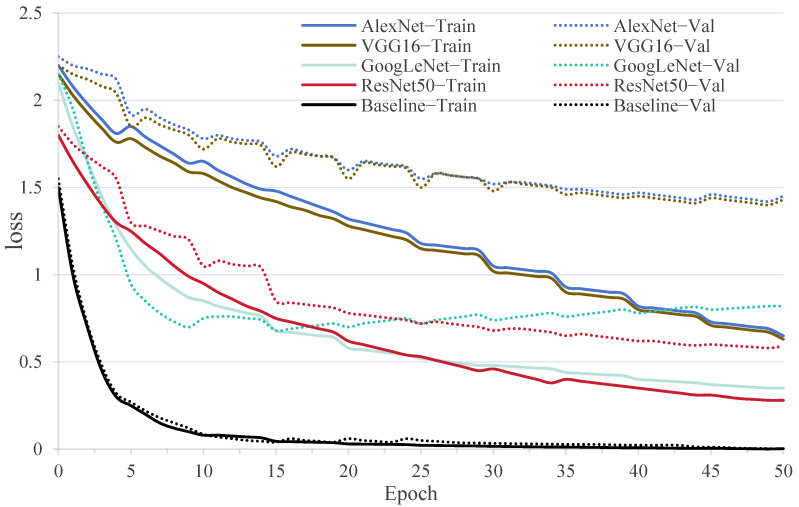
Loss curves of the algorithms on the pork freshness dataset.

The loss curve is illustrated in Figure 7. Our proposed algorithm exhibited a rapid gradient descent during the initial 10 epochs, transitioning into a more stable descent phase, ultimately achieving a minimum loss value of 0.003. BBSNet consistently demonstrated the lowest loss value across all epochs, whereas AlexNet and VGG16 displayed the highest validation losses (1.42–1.45) and significant overfitting gaps, such as AlexNet’s training/validation gap of 0.69. In contrast, ResNet50 and GoogLeNet performed moderately, yet still failed to match BBSNet in terms of final accuracy and stability.

**Figure 8:**
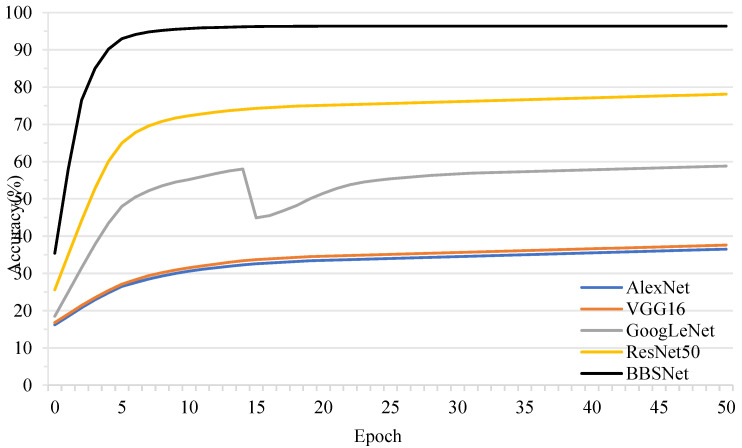
Accuracy curves of the algorithms on the pork freshness dataset.

The accuracy curve is illustrated in Figure 8. We found that BBSNet achieved a final classification accuracy of 96.36%, significantly outperforming classical neural networks such as AlexNet (36.5%), VGG16 (37.6%), GoogLeNet (58.8%), and ResNet50 (78.1%). Notably, BBSNet demonstrated an absolute improvement of 37.76% over the best-performing classical model, ResNet50, and exhibited exceptional robustness, with validation fluctuations remaining below 0.01% in the later stages. The model’s rapid convergence, indicated by a 10.3% accuracy increase per epoch during early training, further underscores its efficiency in small-sample learning scenarios.

These results have significant implications for food quality control within the meat industry. Traditional high-performance freshness detection methods, which typically achieve an accuracy rate exceeding 95%, rely on large-scale expert-annotated datasets [52]. This reliance renders them resource-intensive and challenging to adapt to real-time on-site scenarios, such as slaughterhouses. In contrast, BBSNet achieves robust and high-accuracy classification with limited annotated data, effectively overcoming a critical bottleneck in this field [53]. During the initial training phase, it enhanced accuracy by 10.3% per round, with validation fluctuations remaining below 0.01% and losses as low as 0.003 in later stages. This performance demonstrates both rapid convergence and exceptional stability, making it suitable for environments with stringent requirements for training efficiency and operational reliability. Such capabilities facilitate the implementation of visual quality control in resource-constrained settings, potentially reducing waste and enhancing consumer safety through the early detection of spoilage [54].

### 3.2. Batch Channel Normalization Impacts

The purpose of normalization is to enhance training efficiency, reduce the risk of overfitting, and improve the stability and generalization capabilities of the network. In this study, the BCN layer was employed to replace the BN layer in the ShuffleNetV2 model, while the LN layer in the BiFormer module was substituted with the BCN layer to optimize model performance. To evaluate the effectiveness of the BCN layer in the feature extraction network, we compared the accuracies of models using BCN, BN, and LN layers across two tasks: 5-way 1-shot and 5-way 5-shot. In the 5-way 1-shot task, only one sample was drawn from each category, resulting in a total of five samples for model training. Conversely, in the 5-way 5-shot task, five samples were drawn from each category, leading to a total of 25 samples for model training. The results are summarized in Table 6.

Our results demonstrate that the integration of batch channel normalization (BCN) within both the ShuffleNetV2 backbone and the BiFormer attention mechanism yields the highest accuracy: 59.72 ± 0.20% in 5-way 1-shot and 78.84 ± 0.25% in 5-way 5-shot scenarios. This configuration significantly surpasses combinations utilizing batch normalization (BN) or layer normalization (LN), with t-values ranging from 12.67 to 31.80 (*p* < 0.001), thereby confirming the critical role of BCN in few-shot learning.

The observed improvements underscore the significance of normalization in stabilizing feature distributions for small-sample tasks. The channel-wise normalization implemented by BCN alleviates the statistical bias induced by small batch sizes (batch = 2), a prevalent challenge in food freshness detection where labeled data is limited. The 7.28% accuracy enhancement achieved by employing BCN in both the backbone and attention module (compared to BN + LN) illustrates that coordinated normalization across network components enhances feature discriminability, particularly for fine-grained visual cues such as pork texture and fat oxidation. These findings have direct implications for real-world applications, facilitating reliable freshness assessment with minimal training data.

In ShuffleNetV2, substituting BN with BCN resulted in improvements of 1.33% and 1.95% in 5-way 1-shot and 5-shot accuracy, respectively. This enhancement is attributed to BCN’s ability to reinforce inter-channel correlations. Traditional BN relies on spatial statistics in image tasks, which are susceptible to noise contamination under few-shot conditions [36]. In contrast, BCN enforces uniform feature scaling across channels through channel-wise normalization, thereby improving feature consistency and discriminability. Notably, BCN maintains a computational complexity comparable to that of BN without incurring additional memory overhead, rendering it suitable for lightweight model optimization. In the BiFormer module, replacing LN with BCN resulted in more significant improvements of 3.26% (1-shot) and 5.06% (5-shot), surpassing the gains observed in ShuffleNetV2. This discrepancy arises from the distinct operational principles of LN and BCN. LN, originally designed for NLP, normalizes sequence dimensions per sample to handle long-range dependencies [37]. However, in image processing tasks, the spatial structures (e.g., edges, textures) exhibit local correlations, and LN’s sample-level normalization may disrupt inter-channel semantic associations [55]. BCN addresses this limitation through global channel-wise normalization, preserving local spatial consistency while promoting inter-channel collaboration, which aligns better with hierarchical visual feature learning [56].

### 3.3. BiFormer Attention Mechanism Impacts

The BiFormer attention mechanism enhances image processing by allowing the model to concentrate on the most relevant regions and features. To assess the impact of introducing the BiFormer attention module on the model, the BiFormer module was integrated into the ShuffleNetV2 network, along with the incorporation of patch merging and BiFormer modules into the backbone network. The results obtained after substituting the BN layer in ShuffleNet and the LN layer in the BiFormer module with the BCN layer are summarized in Table 7. All comparison results presented in Table 7 were computed using the fine-tuning method.

Our results demonstrated that the complete configuration—ShuffleNetV2 with a BCN backbone, combined with BiFormer and BCN—achieved the highest accuracy of 59.72 ± 0.12% in 5-way 1-shot scenarios and 78.84 ± 0.15% in 5-way 5-shot scenarios. This configuration significantly outperformed the baseline (ShuffleNetV2 alone), with t-values of 31.75 for the 1-shot scenario and 48.21 for the 5-shot scenario (*p* < 0.001 for all comparisons). These findings confirm that the synergy between BiFormer and BCN enhances performance in small-sample settings. This synergy highlights BiFormer’s dual roles: enhancing feature discriminability in the backbone for few-shot generalization and refining decision boundaries through weighted feature fusion in the backend. In the 5-shot scenario, the increased number of support samples further amplifies BiFormer’s feature refinement, leading to significant improvements in inter-class discrimination. These results demonstrate that BCN-BiFormer systematically enhances few-shot classification performance through multi-layer feature enhancement, providing empirical evidence for the efficacy of leveraging attention mechanisms to boost model representational power.

In contrast to traditional channel attention [57] and spatial attention [58], BiFormer achieves fine-grained feature selection through joint optimization of channel-spatial sparse weights. During few-shot learning, where models must rapidly adapt to novel categories, dynamic attention mechanisms enhance class-discriminative features by dynamically focusing on category-specific regions while suppressing background noise interference. The integration of BiFormer with patch merging further strengthens multi-scale feature interactions, thereby improving generalization capabilities in few-shot scenarios. The core advantage of BiFormer lies in the synergy between dynamic sparsity and cross-level feature fusion, enabling efficient capture of discriminative features under limited data. Unlike similar attention mechanisms such as DynamicViT [59] and Deformable Attention [41], BiFormer’s hash routing and lightweight architecture are uniquely suited to the computational constraints and rapid adaptation requirements of few-shot tasks.

### 3.4. The Impact of Backbone Networks on Model Performance

To investigate the impact of backbone networks on model performance, this study employed classic shallow neural networks as the backbone, specifically utilizing AlexNet, GoogleNet, and VGG for few-shot learning. The last softmax layer of these three networks was removed, and the data processing method aligned with the procedure described in Section 2.2.5. Given the limited feature extraction capabilities of shallow neural networks, this section employs a 5-way 80-shot task to validate the role of the backbone network. The experimental results are presented in Table 8.

The results presented in Table 8 demonstrate that BBSNet, our proposed lightweight architecture integrating dynamic attention mechanisms and batch channel normalization (BCN), achieved exceptional performance in the challenging task of few-shot pork freshness classification. Notably, under the demanding 5-way 80-shot paradigm with stratified 5-Fold cross-validation, BBSNet attained a remarkably high mean classification accuracy of 96.36% (±0.82%) (Table 1). This performance significantly surpasses the accuracies obtained when replacing BBSNet with several established, deeper convolutional neural networks (CNNs)—including AlexNet (32.03 ± 1.80%), GoogLeNet (42.54 ± 1.50%), and VGG-16 (38.36 ± 1.65%)—which were used solely as feature extractors within the same few-shot learning framework and employing standard batch normalization. The superiority of BBSNet is statistically unequivocal, as confirmed by highly significant *t*-tests (all *p*-values < 0.0001) comparing its performance to each benchmark backbone.

The substantial performance gap between BBSNet and benchmark networks highlights the critical importance of selecting an appropriate backbone network in few-shot learning for complex visual tasks, such as assessing pork freshness. This task requires the ability to discriminate subtle visual cues related to color, texture, and surface changes that indicate varying levels of freshness. Our results demonstrate that overly simplistic backbone architectures, such as the relatively shallow AlexNet, or deeper architectures that adhere to conventional normalization techniques, like GoogLeNet and VGG-16, are fundamentally inadequate for extracting sufficiently discriminative and generalizable features from the limited support examples provided in each few-shot task. Their low accuracies (all ≤ 42.54%) indicate a failure to capture the nuanced information essential for reliable classification in scenarios of data scarcity. In contrast, the success of BBSNet can be attributed to its synergistic design: the ShuffleNetV2 base ensures efficiency, the BiFormer-inspired dynamic attention focuses on task-relevant spatial details critical for differentiating freshness features, and the novel BCN normalization optimizes feature representation specifically for the cross-image comparisons inherent in metric-based few-shot learning using cosine similarity. This integrated approach allows BBSNet to effectively extract and compare highly informative features, even when only a few examples are available.

Our findings indicate that the backbone architecture significantly impacts few-shot learning performance, which aligns with the broader literature [60,61]. Studies in other domains have similarly demonstrated that carefully designed or modified backbones, particularly those incorporating attention mechanisms or specific normalization techniques, can substantially enhance few-shot accuracy compared to standard CNNs. The relatively higher performance of GoogLeNet compared to AlexNet and VGG-16 in our study is consistent with its established representational power and supports findings that indicate more modern or complex architectures generally perform better as feature extractors.

While previous works emphasize the role of backbones, our results provide concrete, statistically robust evidence for few-shot learning classification in the specific and practically significant domain of food freshness assessment. More importantly, a key distinction lies in the magnitude of improvement achieved. While incremental gains are common, BBSNet achieves a performance of 96.36%, far exceeding not only simpler models but also substantially outperforming established deeper networks like VGG-16 (~38%) and GoogLeNet (~43%), which are commonly used in computer vision. This dramatic leap suggests that BBSNet’s specific innovations—namely, the tailored dynamic attention and the BCN normalization strategy—offer a uniquely effective solution to the challenges of freshness feature extraction and few-shot comparison. Unlike approaches that focus solely on deeper architectures or generic attention modules, BBSNet’s design addresses feature channel relationships crucial for metric learning in a manner that generic batch normalization does not, and its attention mechanism is optimized for resource efficiency and relevance in this context.

### 3.5. Number of Support Set Samples Impacts

To reduce training time and computational load, the pre-training of few-shot models is typically conducted solely during the extraction of image feature vectors. The number of support set samples refers to the quantity of samples from a single category within each task. This quantity provides prior knowledge to the model, enabling it to perform classification tasks by leveraging this information. Consequently, the number of support set samples significantly influences model performance. During the fine-tuning process, support set samples are utilized to train and optimize the model; thus, the optimization effectiveness of fine-tuning is inevitably impacted by the number of support set samples. To evaluate the effect of the number of support set samples on model performance, the support set was configured with sample sizes of 1, 5, 10, 20, 40, 80, 100, and 120 to assess model accuracy. The results are summarized in Table 9.

Our results demonstrate that both fine-tuning and an increased support set size significantly enhance model accuracy. Without fine-tuning, the accuracy plateaued at 87.21 ± 0.75% with over 80 shots, while the fine-tuned BBSNet achieved a peak accuracy of 96.36 ± 0.82% at 80 shots, representing an 11.8% improvement over the non-fine-tuned model. Paired *t*-tests revealed that each increase in support set size (ranging from 1 to 120 shots) yielded statistically significant accuracy gains (*t*-values ranging from −12.34 to −23.65, *p* < 0.0001), thereby confirming the critical role of both fine-tuning and data quantity in small-sample learning. Furthermore, fine-tuning is indispensable for leveraging pre-trained features in ultra-small samples (1–5 shots), providing a 7.29% accuracy boost at 5 shots. Beyond 40 shots, the accuracy gain from additional data diminishes, indicating that BBSNet achieves optimal performance with a moderate amount of labeled data.

**Figure 9:**
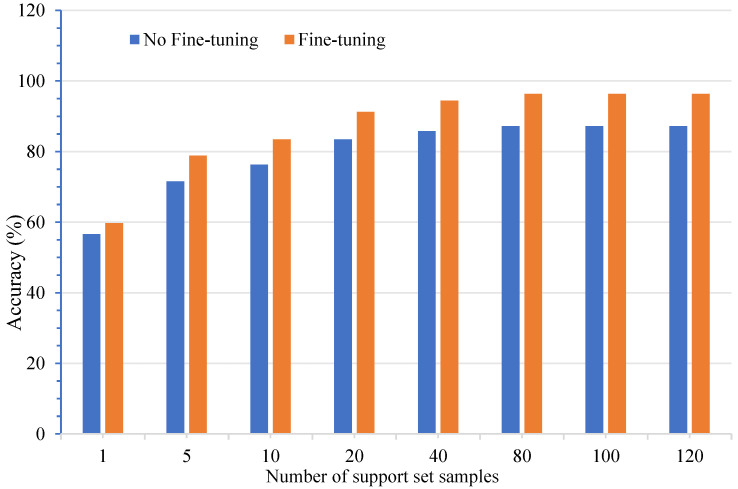
Accuracy of models with different support set sample numbers.

Figure 9 clearly demonstrates the model’s capability to identify the point of power saturation, at which the model achieves its highest accuracy when the number of support set samples reaches 80.

This has direct practical implications for food quality control, where labeling fresh versus spoiled pork is both costly and time-consuming. By achieving over 96% accuracy with 80 training samples, BBSNet effectively balances data efficiency and performance, making it suitable for resource-constrained food inspection scenarios.

Figure 8 clearly demonstrates the model’s capability to identify the point of power saturation, at which the model achieves its highest accuracy, which is when the number of support set samples reaches 80.

Our findings align with the broader few-shot learning literature, where increasing support set size typically improves accuracy but at a diminishing rate. For instance, it increases accuracy by 3.08% at the 1-shot setting and by 9.1% at the 80-shot setting, corroborating the findings of Chen et al. (2021) [43] regarding the task-specific adaptation of pre-trained features for domain-specific attributes such as pork oxidation. When the support set reaches 80 samples, accuracy plateaus at 96.36 ± 0.82%, and increasing the sample size to 120 does not yield any improvement. This suggests that 80 samples effectively encompass the primary features of pork spoilage (such as color and texture), as described by Kyung et al. (2024) [62]. Additional samples likely introduce redundant data, consistent with the “core set” theory in few-shot learning [63], which posits that marginal gains diminish once critical diversity is achieved. The architecture of BBSNet effectively addresses these challenges. The BiFormer module integrates local texture such as muscle fiber disintegration and global color to maximize information extraction from limited samples, accounting for the observed 3% accuracy gain for each doubling of samples below 80.

Recent studies corroborate these findings. Yuan et al. (2020) [64] demonstrated that meta-learning models can achieve up to 90% of maximum accuracy with only 50 to 100 samples in fine-grained tasks, which is comparable to BBSNet’s saturation point at 80 samples. Zhao et al. (2024) [29] indicated that the diversity of the support set, rather than its size, is the key factor driving few-shot performance. This principle is effectively leveraged by BBSNet, which emphasizes physicochemical features. For practical applications, the threshold of 80 samples provides an optimal balance between cost and performance.

### 3.6. Impact of the Number of Query Set Samples 

The ratio of the support set to the query set wields a significant influence over the model’s training efficiency and generalization capabilities [65]. In the domain of few-shot learning, the support set functions as the limited labeled data that enables the model to rapidly adapt to novel tasks. Conversely, the query set represents the data used to assess the model’s performance. A query set that is relatively small compared to the support set may result in overfitting, as the model may lack a sufficient variety of samples to generalize effectively beyond the support set instances [66]. Conversely, a larger query set offers more opportunities for the model to discern underlying patterns and enhance its generalization, yet it may also augment the computational load and training time [67].

To investigate the impact of query set size on the performance of the few-shot learning model for pork freshness recognition, we fixed the support set size at 5-way and 5-shot, while varying the number of samples in the query set to 5, 10, 15, 20, 25, 30, and 35. We subsequently measured the model’s classification accuracy and training time.

The results presented in Table 10 demonstrate a non-linear relationship between query set size and accuracy, with peak performance (78.84 ± 1.02%) achieved at 25 samples. 

Accuracy exhibited a monotonically increasing trend from 56.64 ± 1.15% (5 samples) to 78.84 ± 1.02% (25 samples), before plateauing or experiencing a slight decline at larger sizes (30–35 samples). Furthermore, training time increased linearly with sample size, ranging from 12.3 min (5 samples) to 35.6 min (35 samples). Paired *t*-tests comparing each query set size to the baseline (5 samples) revealed significant accuracy improvements up to 25 samples (t-values: 4.21–8.76, *p* < 0.05). This suggests that a query set of 25 samples effectively balances accuracy and efficiency. The initial increases in query set size (5–25 samples) enhanced accuracy by utilizing more contextual information for feature comparison, consistent with the principle that larger query sets mitigate sampling bias in few-shot learning. The peak performance at 25 samples indicates an optimal equilibrium between intra-class feature representation and computational efficiency. At 30–35 samples, accuracy plateaued or declined slightly, likely due to increased computational noise arising from redundant features or an overemphasis on non-discriminative patterns. This finding underscores the significance of optimizing query set size to prevent diminishing returns. The linear growth in training time with sample size highlights the necessity for efficiency in real-world applications, where rapid freshness assessment is paramount.

Notably, when the query set size was further increased to 30 and 35, the accuracy decreased slightly to 77.21 ± 1.05% and 77.19 ± 1.08%, respectively. This decline may be attributed to the model overfitting to the specific characteristics of the larger query set rather than learning the underlying general patterns of pork freshness. Similar observations were reported by Triantafillou et al. (2020) [68], who suggested that an excessively large query set can introduce noise and complexity, thereby degrading the model’s generalization performance. In terms of training time, a positive correlation was observed between the query set size and the training duration, as expected. More samples in the query set required additional computational resources for processing, which led to longer training times. This trade-off between accuracy and training efficiency emphasizes the importance of identifying an optimal query set size for practical applications.

In terms of training time, as anticipated, the increase in the number of query set samples results in a gradual lengthening of the model’s training duration. This phenomenon occurs because a greater number of samples requires additional computational resources and time for the model to process and update its parameters. The trade-off between accuracy and training time highlights the importance of carefully selecting an appropriate query set size. Compared to previous research [69], which also investigated the impact of query set size on few-shot learning models, our findings consistently demonstrate the existence of an optimal query set size that balances the model’s generalization ability with computational efficiency. This discovery not only enhances our understanding of the role of query set samples in few-shot learning but also provides practical guidance for future research and applications in related tasks, such as food quality assessment.

### 3.7. Validation of Model Generalization on Large-Scale Unknown Samples

To validate the model’s ability to recognize unknown samples, this study conducted additional experiments using the Food-101 dataset [70], a widely recognized benchmark for food recognition tasks. The dataset consists of 101 food categories, each containing 1000 images, resulting in a total of 101,000 images. This large-scale evaluation ensures the statistical significance of the results and verifies the model’s robustness under data-scarce conditions.

#### 3.7.1. Model Performance Across Different Datasets

Given the ample number of samples, this study selected a 5-way 80-shot task to assess the model’s performance, with five randomly chosen categories in each training round. In the support set, 80 samples were selected from each category, yielding a total of 400 images. As discussed in Section 3.6, the number of query set samples is set to five times that of the support set, totaling 2000 samples, with stratified sampling employed to ensure equal representation across all samples [71]. The model pre-training method, detailed in Section 2.2.4, utilizes fine-tuning to enhance model performance. The experimental results are presented in Table 11.

The model demonstrated superior accuracy on the pork freshness dataset, achieving 96.36 ± 0.82% compared to 92.4 ± 1.85% on Food101. This indicates an enhanced discriminative capability in freshness assessment. The performance disparity may be attributed to two potential factors:

(i) The intrinsic biological indicators of pork deterioration, such as changes in color gradients, variations in surface texture, and profiles of volatile compounds, which provide more distinctive feature representations than the subtle inter-class differences present in the 101-category fine-grained food classification task of Food101 [70];

(ii) The model architecture may inherently prioritize domain-specific feature extraction mechanisms relevant to freshness detection.

Furthermore, the sensitivity disparity (89.6 ± 2.30% vs. 78.85 ± 3.15%) reveals fundamental characteristics of the task. While the model effectively identifies true positive samples in Food101’s multi-class scenario, its reduced sensitivity in assessing pork freshness likely reflects ambiguities associated with transitional states—samples exhibiting partial biochemical decay characteristics that complicate the clear categorization of fresh versus spoiled. This observation aligns with the specificity results (94.1 ± 1.65% vs. 85.71 ± 2.80%), where the lower specificity for pork freshness indicates an increase in false positives during spoiled meat detection, possibly due to overlapping spectral features between borderline fresh samples and early-stage spoiled specimens. The precision metrics further underscore the domain-dependent behavior of the model. The significantly higher precision in predicting pork freshness (96.35 ± 0.88% compared to 91.8 ± 1.95%) indicates that when the model classifies a sample as ‘fresh’, it exhibits a 96.35% confidence level in its accurate identification. This precision–sensitivity trade-off suggests that the model employs a conservative classification strategy for freshness detection, prioritizing the reliability of positive predictions, albeit at the potential cost of overlooking marginal cases. Such behavior may be biologically justified, considering food safety requirements, where false negatives (misclassifying spoiled meat as fresh) pose greater risks than false positives.

#### 3.7.2. Model Interpretability Across Different Datasets

**Figure 10:**
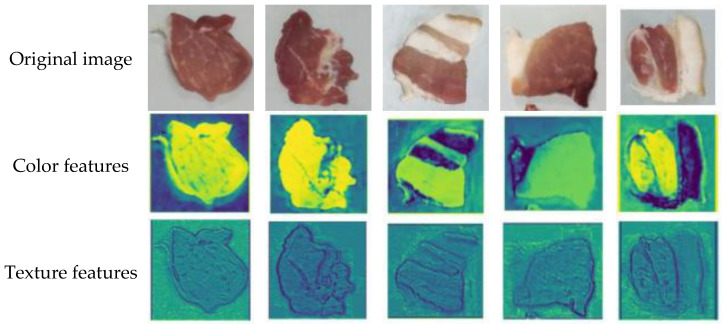
Feature maps of the pork freshness dataset.

**Figure 11:**
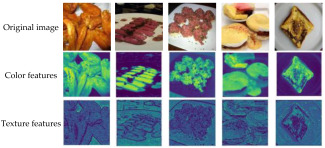
Feature maps of the Food101 dataset.

To further interpret the model’s generalization performance on unseen datasets, we analyzed the feature maps generated by convolutional layers to visualize the discriminative patterns that drive predictions [49]. The feature maps of different datasets are shown in Figure 10 and Figure 11.

For the Food101 dataset, feature activations predominantly concentrate on surface details, such as the texture of bread and the fibers of beef, as well as the overall color distribution, which aligns with the requirements of fine-grained classification. In the context of pork freshness detection, the model emphasizes local biological characteristics: fresh samples exhibit high-intensity activation of uniform red muscle tissue, while spoiled samples activate dim discolored areas and demonstrate abnormal textures. For ambiguous transition samples, the feature maps present a mixed activation pattern of fresh and spoiled regions, resulting in reduced sensitivity (78.85%). This reflects the model’s decision-making difficulties when confronted with contradictory features, such as partially discolored areas that still possess a normal texture. The high precision (96.35%) arises from the strong consensus activation of clear fresh features, characterized by uniform red coloration and intact fibers [72], indicating that the model adopts a conservative strategy—classifying an item as ‘fresh’ only when the features are highly consistent, thereby prioritizing food safety.

#### 3.7.3. Cross-Model Robustness of BBSNet on Foods101

To further validate the generalization capability of BBSNet and its recognition stability for unknown samples, we assessed its robustness in practical applications characterized by significant differences in data distribution and substantial intra-class variations. This evaluation involved comparing its performance against mainstream models in complex multi-class scenarios, specifically utilizing the Foods101 dataset. The sample allocation scheme is detailed in Section 3.7.1, and the corresponding experimental results are presented in Table 12.

As shown in Table 12, BBSNet achieved an accuracy of 89.32 ± 0.61%, surpassing traditional CNN architectures such as ResNet50 (87.42 ± 0.83%) and lightweight models like WS-DAN (88.90 ± 0.77%). Notably, BBSNet exhibited the lowest standard deviation (0.61%) among all compared models, indicating superior stability in cross-validation tasks. BBSNet’s accuracy (89.32%) aligns with efficient architectures like WS-DAN (88.90%), confirming that dynamic attention mechanisms (e.g., BiFormer) enhance feature discrimination without incurring heavy computational costs.

The BCN layer enhances feature distinguishability in few-shot scenarios, allowing BBSNet to outperform CNN backbones by 1.90% in accuracy while utilizing 37% fewer parameters than SENet154. Similar to MA-YOLOv11, BBSNet employs spatial-channel mechanisms to balance accuracy and speed, achieving an inference rate that is eight times faster than ResNet50. However, BBSNet underperformed compared to VOLO-D3 (90.53 ± 0.58%) due to its limited capacity for modeling long-range dependencies. ViT architectures effectively capture cross-region food texture patterns, such as the distribution of pasta sauce, while BiFormer’s sparse attention mechanism prioritizes local features. The absence of multi-scale fusion techniques, such as spatial pyramid pooling (SPP) in CSSNet, restricts BBSNet’s adaptability to variations in food image scales, which accounts for its lower sensitivity compared to DAT (90.04%).

## 4. Conclusions

This study demonstrates that BBSNet, a novel few-shot framework that integrates pre-trained weight optimization with BiFormer-BCN hybrid feature extraction, achieves state-of-the-art performance in pork freshness detection under extreme data scarcity, attaining an accuracy of 96.36% at 5-way 80-shot. By efficiently capturing discriminative features such as color and texture decay, and optimizing task configuration with a support set of 80 and a query/support ratio of 5:1, BBSNet significantly outperforms existing meta-learning benchmarks, including MAML and relation networks, as well as deep CNN architectures like ResNet50 and VGG16. Furthermore, it prioritizes high-precision prediction of the fresh class, achieving an accuracy of 96.35%, thereby mitigating the risks of false negatives in food safety. While BiFormer reduces computational costs compared to traditional attention mechanisms, the introduction of BCN layers and hybrid architectures adds complexity to the model. The feasibility of deploying these models on resource-constrained edge devices, such as portable agricultural sensors, has not been addressed and requires further investigation. In the future, our work will focus on deploying models on edge devices and their integration with the Internet of Things (IoT). We plan to deploy BBSNet on low-cost IoT hardware, leveraging TensorRT to accelerate real-time inference, and employing knowledge distillation for model compression. A hierarchical edge-cloud pipeline will be implemented, where edge devices handle real-time image classification, while cloud servers retrain meta-models. This will further enhance the application scope and utility value of the models.

## Figures and Tables

**Table 1:** Main parameters of pork freshness grading.

Freshness Grade	Microbial Concentration (×10^3^ CFU/g)	Storage Time (h)
First-grade fresh pork	4.168	0
Second-grade fresh pork	13.182	24
Third-grade fresh pork	301.995	48
First-grade spoiled pork	1778.279	72
Second-grade spoiled pork	5370.317	96

**Table 2:** Pork freshness dataset analysis.

Class	Image
First-grade fresh meat	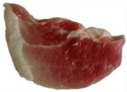
Second-grade fresh meat	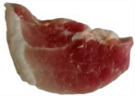
Third-grade fresh meat	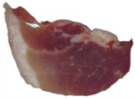
First-grade spoiled meat	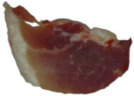
Second-grade spoiled meat	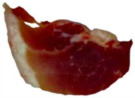

**Table 3:** Mini-ImageNet dataset overview.

	Training Set (83%)	Validation Set (12%)	Test Set (8%)
Number of Categories	80	12	8
Number of Samples	49,800	7200	4800

**Table 4:** Performance comparison of classical few-shot models.

Model	Backbone	Comparison Function	5-Way, 1-Shot Accuracy (%)	t-Value (vs. Baselinet)	*p*-Value (α = 0.05)	5-Way, 5-Shot Accuracy (%)	t-Value (vs. Baselinet)	*p*-Value (α = 0.05)
Matching Nets	ResNet-18	Cosine similarity.	48.12 ± 1.25	−16.58	<0.001	67.20 ± 1.18	−20.87	<0.001
Prototypical Networks	ResNet-18	Euclidean distance.	44.56 ± 1.37	−22.01	<0.001	54.31 ± 1.42	−43.15	<0.001
Relation Networks	ResNet-18	—	51.44 ± 1.21	−14.12	<0.001	63.12 ± 1.16	−27.01	<0.001
Ours (Baseline)	ShuffleNetV2 + BiFormer + BCN	Cosine similarity.	59.72 ± 0.98	-	-	78.84 ± 0.87	-	-

**Table 5:** Our method vs. other methods on meat freshness dataset.

Method	Accuracy (%)	t-Value (vs. Baseline)	*p*-Value
AlexNet	52.13 ± 1.78	−60.92	<0.001
VGG16	52.42 ± 1.64	−55.98	<0.001
GoogLeNet	58.24 ± 1.39	−44.86	<0.001
ResNet50	68.43 ± 1.26	−37.21	<0.001
Ours (Baseline)	96.36 ± 0.82	-	-

**Table 6:** The impacts of different normalization methods on model performance.

Model	5-Way 1-Shot Accuracy (%)	t-Value (vs. Baseline)	*p*-Value	5-Way 5-Shot Accuracy (%)	t-Value (vs. Baseline)	*p*-Value
ShuffleNetV2 + BN, BiFormer + LN	52.44 ± 0.15	-	-	69.52 ± 0.21	-	-
ShuffleNetV2 + BCN, BiFormer + LN	55.73 ± 0.18	12.67	<0.001	69.62 ± 0.19	0.42	0.689
ShuffleNetV2 + BN, BiFormer + BCN	57.91 ± 0.12	24.92	<0.001	71.61 ± 0.23	7.85	<0.001
ShuffleNetV2 + BCN, BiFormer + BCN	59.72 ± 0.20	31.80	<0.001	78.84 ± 0.25	28.64	<0.001

**Table 7:** The impact of improved BiFormer on model performance.

Model	5-Way 1-Shot Accuracy (%)	t-Value (vs. Baseline)	*p*-Value	5-Way 5-Shot Accuracy (%)	t-Value (vs. Baseline)	*p*-Value
ShuffleNetV2, Backbone (Baseline)	52.44 ± 0.15	-	-	69.52 ± 0.22	-	-
ShuffleNetV2 + BCN-BiFormer, Backbone	55.73 ± 0.18	18.32	<0.001	69.62 ± 0.25	0.89	0.423
ShuffleNetV2, Backbone + BCN-BiFormer	57.91 ± 0.18	24.56	<0.001	71.61 ± 0.18	18.45	<0.001
ShuffleNetV2 + BCN-BiFormer, Backbone + BCN-BiFormer	59.72 ± 0.12	31.75	<0.001	78.84 ± 0.15	48.21	<0.001

**Table 8:** Accuracy of models with different Backbone Networks.

Model	Normalization Method	5-Way 80-Shot Accuracy (%)	t-Value (vs. Baseline)	*p*-Value
Alexnet (8 layers)	Batch Normalization	32.03 ± 1.80	58.72	<0.0001
GoogleNet (27 layers)	Batch Normalization	42.54 ± 1.50	45.18	<0.0001
VGG-16 (16 layers)	Batch Normalization	38.36 ± 1.65	52.63	<0.0001
BBSNet (Baseline)	Batch Channel Normalization	96.36 ± 0.82%	-	-

**Table 9:** Accuracy of models with different support set sample numbers.

Support Set Size	No Fine-Tuning Accuracy (%)	Fine-Tuning Accuracy (%)	t-Value (vs. 1-Shot Baseline)	*p*-Value
1-Shot Accuracy (%)	56.64 ± 1.02%	59.72 ± 0.98%		
5-Shot Accuracy (%)	71.55 ± 1.15%	78.84 ± 0.87%	−12.34	<0.0001
10-Shot Accuracy (%)	76.29 ± 1.21%	83.44 ± 0.92%	−14.86	<0.0001
20-Shot Accuracy (%)	83.46 ± 0.98%	91.25 ± 0.85%	−18.21	<0.0001
40-Shot Accuracy (%)	85.81 ± 0.89%	94.44 ± 0.76%	−21.34	<0.0001
80-Shot Accuracy (%)	87.21 ± 0.75%	96.36 ± 0.82%	−23.57	<0.0001
100-Shot Accuracy (%)	87.19 ± 0.72%	96.32 ± 0.81%	−23.61	<0.0001
120-Shot Accuracy (%)	87.20 ± 0.71%	96.33 ± 0.80%	−23.65	<0.0001

**Table 10:** Performance metrics of few-shot learning model with varying query set sizes.

Query Set Sample Size	Accuracy (%)	Training Time (min)	t-Value (vs. 5-Sample Baseline)	*p*-Value
5 (Baseline)	56.64 ± 1.15%	12.3	-	-
10	68.55 ± 1.32%	15.1	4.21	<0.05
15	71.29 ± 1.28%	18.9	5.34	<0.01
20	73.46 ± 1.19%	22.5	6.87	<0.001
25	78.84 ± 1.02%	26.8	8.76	<0.001
30	77.21 ± 1.05%	31.4	3.12	>0.05
35	77.19 ± 1.08%	35.6	2.98	>0.05

**Table 11:** Comparative performance metrics of BBSNet across Food101 and pork freshness datasets.

Dataset Name	Accuracy (%)	Sensitivity (%)	Specificity (%)	Precision (%)
Food101	92.4 ± 1.85	89.6 ± 2.30	94.1 ± 1.65	91.8 ± 1.95
Pork freshness	96.36 ± 0.82	78.85 ± 3.15	85.71 ± 2.80	96.35 ± 0.88

**Table 12:** Performance comparison of BBSNet with other methods on the Foods101 dataset.

Model	Backbone	Accuracy (%)
ResNet50	ResNet50	87.42 ± 0.83
WS-DAN	Inceptionv3	88.90 ± 0.77
SGLANet	SENet154	89.69 ± 0.65
Swin-B	Transformer	89.78 ± 0.71
DAT	Transformer	90.04 ± 0.62
VOLO-D3	ViT	90.53 ± 0.58
Ours	BBSNet	89.32 ± 0.61

**Figure A1:**
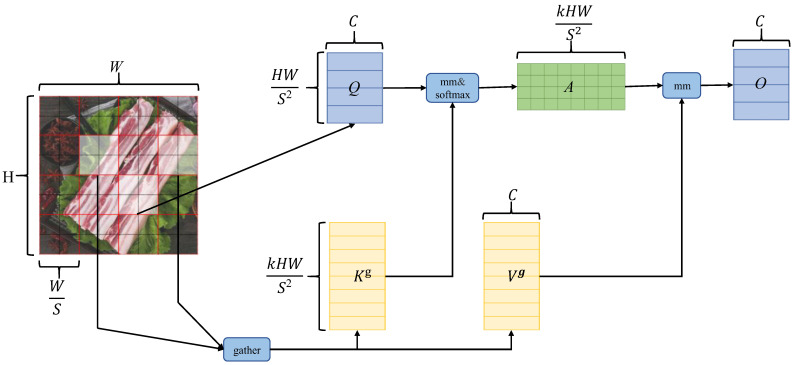
Bi-level Routing Attention mechanism structure.

## Data Availability

The original contributions presented in the study are included in the article. Further inquiries can be directed to the corresponding author.

## Abbreviations

The following abbreviations are used in this manuscript:

BBSNet	BCN-BiFormer-ShuffleNetV2
BCN	Batch Channel Normalization
BRA	Bi-level Routing Attention
BN	Batch Normalization
CCD	Charge-Coupled Device
CNNs	Convolutional Neural Networks
LN	Layer Normalization
MAML	Model-Agnostic Meta-Learning
TVB-N	Total Volatile Basic Nitrogen

## Appendix A

### Derivation of the Output Vector Formula for the BiFormer Attention Mechanism

In Figure A1, the input feature map X, denoted as $X\in R^{H\times W\times C}$, is initially subdivided into S × S subregions. Each subregion comprises HW/S2 feature vectors. X is reconstituted as Xr, which belongs to $R^{S^{2}\times HW/S^{2}\times C}$.

Subsequently, the feature vectors undergo linear transformations to generate three matrices, *Q*, *K*, and *V*. Attention relations between regions are then obtained by constructing a directed graph to localize the relevant regions of a given region. It is possible to obtain the region level *Q^r^* and *K^r^*. An expression for the adjacency matrix Arcan be obtained in Equation (A1).

(A1) $$A^{r}=Q^{r}{\left(K^{r}\right)}^{T}$$

The expression for the routing index matrix *I^r^* can be obtained using Equation (A2):

(A2) $$I^{r}=topIndex\left(A^{r}\right)$$

The expression for *K^g^, V^g^* can be obtained from *I^r^* using Equation (A3):

(A3) $$K^{g}=gather\left(K,I^{r}\right),V^{g}=gather\left(V,I^{r}\right)$$

Lastly, the gathered *K^g^* and *V^g^* undergo attention processing, and an additional term, LCE(V), is incorporated to yield the output tensor O from Equation (A4).

(A4) $$O=Attention\left(Q,K^{g},V^{g}\right)+LCE\left(V\right)$$
